# High Rates of Non-Tuberculous Mycobacteria Isolation in Mozambican Children with Presumptive Tuberculosis

**DOI:** 10.1371/journal.pone.0169757

**Published:** 2017-01-17

**Authors:** Elisa López-Varela, Alberto L. García-Basteiro, Orvalho J. Augusto, Oscar Fraile, Helder Bulo, Tasmiya Ira, Kizito Gondo, Jakko van Ingen, Denise Naniche, Jahit Sacarlal, Pedro L. Alonso

**Affiliations:** 1 Centro de Investigação em Saude de Manhiça (CISM), Maputo, Mozambique; 2 ISGlobal, Barcelona Ctr. Int. Health Res. (CRESIB), Hospital Clínic de Barcelona, Universitat de Barcelona, Barcelona, Spain; 3 Amsterdam Institute for Global Health and Development (AIGHD), Amsterdam, The Netherlands; 4 Department of Medical Microbiology, Radboud University Medical Center, Nijmegen, The Netherlands; 5 Departamento de Microbiologia, Faculdade de Medicina, Universidade Eduardo Mondlane, Maputo, Mozambique; Hopital Raymond Poincare - Universite Versailles St. Quentin, FRANCE

## Abstract

**Introduction:**

Non-tuberculous mycobacteria (NTM) can cause disease which can be clinically and radiologically undistinguishable from tuberculosis (TB), posing a diagnostic and therapeutic challenge in high TB settings. We aim to describe the prevalence of NTM isolation and its clinical characteristics in children from rural Mozambique.

**Methods:**

This study was part of a community TB incidence study in children <3 years of age. Gastric aspirate and induced sputum sampling were performed in all presumptive TB cases and processed for smear testing using fluorochrome staining and LED Microscopy, liquid and solid culture, and molecular identification by GenoType^®^ Mycobacterium CM/AS assays.

**Results:**

NTM were isolated in 26.3% (204/775) of children. The most prevalent NTM species was *M*. *intracellulare* (N = 128), followed by *M*. *scrofulaceum* (N = 35) and *M*. *fortuitum* (N = 9). Children with NTM were significantly less symptomatic and less likely to present with an abnormal chest radiograph than those with *M*. *tuberculosis*. NTM were present in 21.6% of follow-up samples and 25 children had the same species isolated from ≥2 separate samples. All were considered clinically insignificant and none received specific treatment. Children with NTM isolates had equal all cause mortality and likelihood of TB treatment as those with negative culture although they were less likely to have TB ruled out.

**Conclusions:**

NTM isolation is frequent in presumptive TB cases but was not clinically significant in this patient cohort. However, it can contribute to TB misdiagnosis. Further studies are needed to understand the epidemiology and the clinical significance of NTM in children.

## Introduction

Non-tuberculous mycobacteria (NTM) are a large family of acid-fast bacteria, widespread in the environment and common in soil and water [1]. In children, the most frequent NTM disease is cervicofacial lymphadenitis, followed by skin and soft tissue infections. It can occasionally produce lung disease and disseminated infection, although the latter are extremely rare in the absence of genetic disorders (cystic fibrosis and mendelian susceptibility to mycobacterial disease) or acquired immunodeficiency [2–4].

Childhood TB is a frequent cause of lung disease in high TB endemic countries[5]. However, underdetection is common [6], partially due to the inherent difficulties in obtaining respiratory samples in children, coupled to the paucibacillary nature of TB in this age group [5]. An additional layer of complexity is posed by the fact that NTM lung disease and tuberculosis (TB) have overlapping clinical and radiological manifestations. This poses a diagnostic and therapeutic challenge in high TB settings where sophisticated laboratory services are unavailable[7]. Moreover, it has been suggested that NTM may have an effect on the response to BCG vaccination and a better understanding of this association is needed in the context of novel tuberculosis vaccine assessment[8].

The epidemiology of NTM varies by world region, however there are few studies reporting NTM isolation in the pediatric population, especially in low resource TB endemic settings[9–11]. Little data is available on the epidemiology and clinical burden of this neglected cousin of TB. The objective of this study is to determine the prevalence and describe the clinical characteristics associated with NTM isolation in young children in a rural area of Southern Mozambique.

## Materials and Methods

### Study setting

This study was conducted at the Manhiça Health Research Center (CISM), located in Southern Mozambique, a semi-rural area with a high burden of HIV and TB[12]. The region is a farming area where 38% of the households have formal housing and 55% are supplied with piped water [13]. The Bacille Calmette-Guerin (BCG) vaccination coverage is above 95% [14].

### Study design

This study was part of a larger prospective descriptive study assessing the minimum community incidence of TB among young children (<3 years of age) over a 1-year period (October 2011–2012)[15]. TB cases were classified according to the NIH classification [16]. This study showed that the incidence rate of TB among children aged less than 3 years was 470/100,000person-years, with a low estimated case detection rate of 40.8% (95% CI 36.6–45.1%)[6,15].

### Clinical procedures

Children with symptoms suspicious of TB and those in close contact with a sputum smear-positive TB case were recruited through active and passive case detection system and evaluated through physical examination, HIV rapid antibody test (Determine^®^, Abbott Laboratories), tuberculin skin test (TST) and a chest radiograph (CXR). For symptomatic cases, upon admission to the study, one induced sputum with nasopharyngeal suction (IS) and one gastric aspirate (GA) same-day ambulatory samples were collected following standard operating procedures from each participant and evaluated by smear microscopy and culture. All participants included had at least one follow up visit arranged within six months of recruitment regardless of initial disease classification in order to assess symptom resolution with or without TB treatment. Those who remained symptomatic were re-assessed, by repeated CXR and collection of new samples. Full details on the study procedures are described elsewhere[15,17]. Clinical management of newly diagnosed TB cases was performed by the National Tuberculosis Program (NTP) according to established national guidelines.

### Laboratory procedures

Sterile single use consumables were used for each clinical procedure and no prior oral cavity decontamination took place. GA was performed placing appropriate-sized nasogastric tube in the stomach of a child with a minimum of 4h fasting. If less than 3ml were retrieved, the stomach was washed with 10 ml of normal saline pushed via NG tube. The sample was neutralized with 2ml bicarbonate on the spot. In children >3 months of age, an additional sample was obtained through sputum induction, which implied administration of 100μg of salbutamol by holding chamber followed by nebulizing 5-7ml of 3% hypertonic saline. A suction catheter was inserted through the nostril into the oropharynx and secretions were aspirated mechanically Samples were transported within 4 hours of collection and processed in the TB laboratory at CISM. The laboratory is subject to an External Quality Assurance program provided by the National Health Laboratory Service in South Africa and is certified by the International Organization for Standardization (ISO 90001:2008 for Quality Management).

Briefly, samples were subject to digestion, decontamination and concentration with NALC/NaOH for 15 minutes, followed by centrifugation (3000g/15min), and resuspension in 2ml of phosphate buffered saline (PBS). Sediments were inoculated into liquid (MGIT) and solid culture media (Lowenstein Jensen; Beckton Dickison (BD) and incubated for 45 and 60 days respectively. Smears were stained with auramine O.

Specifically, the gastric lavage specimens were processed prior to the digestion-decontamination through the addition of 50mg of NALC, centrifugation at 3000 g for 15 minutes and resuspension of the sediment in 2ml of sterile PBS.

Positive cultures were confirmed using ZN staining and immunochromatographic assay, BD MGIT TBc Identification Test (TBc ID, Becton Dickinson, Sparks, MD) as well as Xpert MTB/RIF (Cepheid, Sunnyvale, CA) and identified using HAIN GenoType^®^ Mycobacterium CM/AS line probe assays.

### Ethical approval

The study protocol was approved by the Mozambican National Bioethics Committee and the Hospital Clinic of Barcelona Ethics Review Committee. Written informed consent was obtained from the caretakers of all study participants.

### Data analysis and statistical considerations

Clinical data was double entered in an electronic data capture system (OpenClinica^™^ www.openclinica.org) and checked for discrepancies. Statistical software for analysis was Stata 13.0 (StataCorp. 2013. Stata: Release 13. Statistical Software. College Station, TX: StataCorp LP).

The prevalence of NTM was calculated as the proportion of NTM positive cultures at initial admission visit over those who underwent bacteriological investigation. Two children had TB isolated at a follow-up visit (N = 2) and were excluded from the follow-up analysis.

## Results

### Culture results

A total of 789 presumptive TB cases were admitted for investigation in the study. Of them, 775 children had at least one mycobacterial culture result available at the initial visit: 11 had a positive culture for MTB (1.4%) and 204 had an NTM isolate (26.2%) (Table 1). The prevalence of NTM isolate findings was 16.0% (118/738) for gastric aspirate and 15.3% (111/726) for induced sputum. The diagnostic yield of liquid culture alone was 15.7% (109/695) for GA and 14.8% (103/698) for IS compared to 3.9% (25/641) for GA and 3.8% (25/662) for IS in solid media alone. The overall rate of sample contamination was larger among GA vs. IS (3.8% vs. 1.8%) and solid vs. liquid culture (13.5% vs. 7.5). Among all presumptive TB cases, seven had a positive smear (3 had an NTM isolated, one did not have culture results available and the remaining three had negative cultures). Gastric aspiration was able to identify a larger number of *M*. *tuberculosis* (MTB) isolates than IS (7 vs. 4). All MTB cases were positive on liquid culture and only 4 of them were also positive on solid media.

**Table 1:** Mycobacterial culture results per sample available at admission.

	Gastric Aspirate (N, %)		Induced Sputum (N, %)	
Performed	773		750	
Smear positive	3	0,39%	6	0,80%
Culture results available	767	99,22%	739	98,53%
**Liquid culture**				
Contaminated	72	9,39%	41	5,55%
Negative	579	75,49%	591	79,97%
NTM	109	15,71%	103	14,76%
MTB	7	1,01%	4	0,57%
**Solid culture**				
Contaminated	126	16,43%	77	10,42%
Negative	615	80,18%	634	85,79%
NTM	25	3,90%	25	3,78%
MTB	1	0,16%	3	0,45%
**Liquid plus solid culture**				
Contaminated	29	3,78%	13	1,76%
Negative	613	79,92%	611	82,68%
NTM	118	15,99%	111	15,29%
MTB	7	0,95%	4	0,55%

Abbreviations: NTM: Nontuberculous mycobacteria; MTB: *M*. *tuberculosis*. Footnote: % of NTM and MTB among uncontaminated samples

Mycobacterial culture yielded 187 identifiable NTM isolates comprising 8 different species and 35 unidentifiable NTM. The most prevalent NTM was *M*. *intracellulare* (N = 128), followed by *M*. *scrofulaceum* (N = 35) and *M*. *fortuitum* (N = 9) (Table 2). NTM were isolated both in GA and IS in 25 children, 9 of them had the same NTM species (all of them were *M*. *intracellulare*). Two or more NTM species were isolated in 18 children; two cases had both isolates identified in the same sample.

**Table 2:** Frequency of different specimens according to sample type.

SAMPLES					PATIENTS (Gastric aspirate plus induced sputum)		
NTM species	Gastric aspirate (N = 118)		Induced sputum (N = 111)		Single isolate	Coinfection	
	N, %		N %		(N)	(N, %)	
M. intracellulare	67	56,8%	70	63,1%	114	14	10,9%
M. scrofulaceum	17	14,4%	18	16,2%	27	8	22,9%
Mycobacterium Sp.	20	16,9%	12	10,8%	28	4	12,5%
M. fortuitum	6	5,1%	3	2,7%	8	1	11,1%
M. malmoense	2	1,7%	3	2,7%	2	3	60,0%
M. interjectum	5	4,2%	0	0,0%	2	3	60,0%
M. gordonae	1	0,8%	1	0,9%	2	0	0,0%
M. chelonae	0	0,0%	3	2,7%	1	2	66,7%
M. abscessus	0	0,0%	1	0,9%	1	0	0,0%

### Clinical characteristics associated with NTM isolation

Among those with NTM isolates, 42.2% were female and 52.5% were between 12 and 23 months of age (Table 3). The prevalence of NTM isolates slightly increased with age (from 22% in the first year to 27% in the third year of life) and a BCG scar was present in 84.3% of the cases. The most frequent clinical feature at enrolment was malnutrition (89.7%) followed by prolonged cough (18.1%). At physical examination, two children had lymphadenopathy (one was cervical and one was a fistulized inguinal node) and 4 had an abnormal lung examination. Fifteen percent had a positive TST and 11.8% were HIV positive. At admission, 35 children had abnormal CXR (17.6%).

**Table 3:** Baseline characteristics of presumptive TB cases with at least one sample at admission according to mycobacterial culture results.

	NTM (N, %)		MTB (N, %)		Univariate	
	204		11		OR (95% CI)	p
**Sex**						
male	118	57,84%	5	45,45%		
female	86	42,16%	6	54,55%	0.53 (0.48–5.59)	0.42*
**Age in months (Median [IQR])**	20.7 (14.9–26.2)		21.4 (14.5–33.6)			
**Age category, N (%)**						
< 1	32	15,69%	2	18,2%	1	
1–2	107	52,45%	5	45,5%	0.75 (0.14–4.06)	
2-+	65	31,86%	4	36,4%	0.98 (0.17–5.71)	0.84*
**BCG Scar**						
Absent	31	15,20%	4	36,4%	1	
Present	172	84,31%	7	63,6%	0.31 (0.09–1.16)	0.08
**TB contact (documented or reported)**						
No	188	92,16%	10	90,9%	1	
Yes	16	7,84%	1	9,1%	1.17 (0.14–9.82)	0.61
**Number of consultations in previous year**						
Median (IQR)						
< 10	179	87,75%	6	54,5%	1	
10 - +	25	12,25%	5	45,5%	5.97 (1.64–21.69)	0.01
**Symptoms at enrollment**						
**Cough ≥2 weeks**						
no	167	81,86%	5	45,5%	1	
Yes	37	18,14%	6	54,5%	5.41 (1.52–19.24)	0.01
**Fever≥2 weeks**						
no	197	96,57%	7	63,6%	1	
yes	7	3,43%	4	36,4%	16.08 (3.48–74.27)	< 0.001
**Chronic or Acute Malnutrition malnutrition**						
no	21	10,29%	5	45,5%	1	
yes	183	89,71%	6	54,5%	0.14 (0.04–0.51)	< 0.001
**Wheeze**						
no	201	98,53%	10	90,9%	1	
yes	3	1,47%	1	9,1%	6.7 (0.62–72.00)	0.07
**Adenopathy**						
no	202	99,02%	10	90,9%	1	
yes	2	0,98%	1	9,1%	10 (0.81–125.30)	0.03
**Number of positive criteria**						
One	164	80,39%	5	45,5%	1	
More than one	40	19,61%	6	54,5%	4.92 (1.39–17.37)	0.006
**Hospitalized during TB presumption**						
no	193	94,61%	8	72,7%	1	
yes	11	5,39%	3	27,3%	6.58 (1.48–29.23)	0.004
**Physical Exam**						
**Abnormal lung exam**						
no	200	98,04%	8	72,7%	1	
yes	4	1,96%	3	27,3%	18.7 (3.26–107.65)	< 0.001
**Fever**						
no	201	99,50%	9	90,0%	1	
yes	1	0,50%	1	10,0%	22.33 (1.20–414.94)	0.002
**Lymphadenoapthy**						
no	202	99,02%	10	90,9%	1	
yes	2	0,98%	1	9,1%	10.1 (0.81–125.30)	0.03
**Tuberculin skin test**						
Negative	173	85,22%	10	90,9%	1	
Positive	30	14,78%	1	9,1%	0.58 (0.07–4.70)	0.6
**HIV Reported**						
Not positive	180	88,24%	9	81,8%	1	
Positive	24	11,76%	2	18,2%	1.66 (0.34–8.21)	0.53
**Radiological changes suggestive of TB**						
No	164	82,41%	2	22,22%	1	
Yes	35	17,59%	7	77,78%	16.4 (3.00–89.60)	< 0.001

Abbreviations: NTM: Nontuberculous mycobacteria; MTB: *M*. *tuberculosis;* OR: odds ratio; IQR: Interquartile range; TB: Tuberculosis.

* = p values calculated using the Fisher's exact test.

Compared to children with NTM isolates, those with MTB were sicker: they had more number of outpatient department visits in the previous year (OR 5.97, 95% CI 1.64–21.69, p = 0.01), presented more symptoms (OR 4.92, 95% CI 1.39–17.37 p = 0.006), and were more likely to present with an abnormal CXR (OR 16.4, 95% CI 3.00–89.60 p<0.001). There were no differences in TST or HIV positivity.

There was no significant difference between the prevalence of the NTM at admission in HIV-infected and uninfected TB presumptive cases (25 vs. 30%, respectively).

### Follow-up data and outcomes

Twenty-five children were identified as having the same NTM species isolated from at least two separate IS or GA samples (all except for two were *M*. *intracellulare*). Three fulfilled the microbiological criteria for diagnosing NTM lung disease[18] and had pulmonary symptoms plus abnormal CXR. However, these isolates were considered clinically insignificant. None of the children were treated for NTM disease, one received TB treatment and there were no deaths registered two years post admission.

We did not find any difference regarding mortality or likelihood of TB treatment initiation in those cases who had an NTM isolated compared to those with a negative culture, supporting the clinical insignificance of NTM isolates (Table 4). However, children with NTM were more likely to be classified as probable or possible TB, more likely to initiate isoniazid preventive therapy (IPT), and time to treatment initiation was shorter.

**Table 4:** Outcomes and follow-up information according to culture result at admission.

Outcomes	NTM (N, %)		MTB (N, %)		Negative (N, %)	
	204		11		567	
TB case type*						
Confirmed	0		11		0	
Probable	7	3,43%	0		25	4,41%
Possible	48	23,53%	0		48	8,47%
MTB infection	24	11,76%	0		21	3,70%
Unlikely TB	125	61,27%	0		473	83,42%
**Isoniazide preventive treatment**						
No	181	88,73%	11	100%	527	92,95%
Yes	23	11,27%	0	0%	40	7,05%
**TB treatment**						
No	194	95,10%	4	36,4%	534	94,18%
Yes	10	4,90%	7	63,6%	33	5,82%
**Median time to treatment initiation (days), N (IQR)**	51 (41–100)		35 (33–63)		140 (48–233)	
**Mortality at 24 months**						
No	190	93,14%	8	72,7%	529	93,30%
Yes	14	6,86%	3	27,3%	38	6,70%

Abbreviations: NTM: Nontuberculous mycobacteria; MTB: *M*. *tuberculosis;* TB: Tuberculosis; IQR: Interquartile range.

* NIH TB case definition

Most patients (88.4%), regardless of the initial culture result (204 NTM, 11 MTB, 567 negative), had at least one follow-up visit. Among them, 14.8% had follow-up samples collected with an overall prevalence of NTM of 21.6%. This number was the same for those children with an NTM compared to a negative sample at admission (22.2 vs. 21.6 respectively).

## Discussion

To our knowledge, this is the first study reporting the rate of NTM isolation in children in Mozambique and one of the few in Sub-Saharan Africa. Our findings suggest that NTM isolation in GA and IS samples of presumptive childhood TB cases is very frequent but may not be clinically significant in this patient cohort. Thus, in high TB endemic countries, NTM isolation complicates patient management as the underlying diagnosis is most often assumed to be TB.

Recent studies in TB endemic countries have reported high rates of NTM isolation in presumptive TB cases[10,19–22]. Adult studies in Nigeria and Uganda have shown a significant increase in NTM isolation rate from 1–4% in older studies using solid media to 15% in more recent ones in which liquid media were used [23]. In our cohort the yield of NTM was significantly higher in liquid than solid culture in agreement with the results of a meta-analysis showing that liquid culture alone has a 66% sensitivity compared with 51% for solid media alone [24]. The single published paper reporting NTM isolates in Mozambique detected NTM isolates in the sputum of 3 out of 320 HIV infected adults [25]. In children tested for tuberculosis, several reports have described frequent NTM isolates both in gastric aspirates and in sputum, ranging from 4 to 10%[9,10,26,27]. Although some authors detected a higher yield of induced sputum versus gastric lavage[10], in our study the rate was similar for both types of samples.

Once isolated in a respiratory sample, it is often difficult to distinguish whether the NTM are causally related to the clinical disease, simply a reflection of recent environmental exposure, or a contaminant in biological specimen related to the sampling technique or laboratory equipment[28]. Environmental exposure to NTM has been hypothesized to increase as children grow older [9,10]; differences in prevalence among children of different age were also observed in the current study, but there was no clear increase with increasing age. In our study, the rate of isolation and the distribution of species did not vary substantially throughout the study period, which renders systematic NTM laboratory contamination unlikely. Although sterile water was used for the sampling procedures, systematic decontamination of the oral cavity was not performed and thus, we cannot rule out the possibility of contamination of the mouth/gastrointestinal tract immediately prior to the sampling process.

A symptomatic child with a positive culture yielding NTM does not indicate pulmonary disease per se [1] and does not necessarily require treatment [10]. In this cohort, we believe that the isolated NTM were not clinically significant. Firstly, none of the children received NTM specific treatment and all 3 symptomatic cases that fulfilled the microbiological and radiological criteria for NTM lung disease were alive and well two years later. Secondly, we observed that the proportion of children with an NTM isolate at a follow-up visit was the same regardless of the initial culture result. Finally, and although we could not compare our results to a control group, the 2 year all-cause mortality did not increase as compared to those children with negative cultures. On the other hand, children with MTB isolates had a worse clinical and radiological presentation and higher two year all-cause mortality than those with NTM. These results are similar to those published by Hatherill et al, who reported that presumptive TB cases with NTM isolates were less likely to demonstrate radiological features compared to MTB. However, they did see an association between NTM and older age, constitutional symptoms and lower rates of positive TST[10].

We did observe that isolating an NTM decreased the odds of ruling out TB according to NIH case definition as compared to children with negative culture at admission (Table 4). Besides, children with NTM isolates were more likely to initiate IPT and although there were no differences in the proportion of children initiating treatment, time to treatment initiation was much shorter if a NTM was found on admission. Without molecular methods, often unavailable in TB endemic countries, a positive sputum smear for NTM is likely to be misinterpreted as tuberculosis. In this study, almost half of the positive smears were due to NTM. These findings highlight that NTM isolates can pose a clinically significant obstacle to the accurate diagnosis of childhood tuberculosis, potentially overdiagnosing the TB cases. This is particularly important when designing TB vaccine trials for two reasons: firstly, because NTM exposure may affect the efficacy of BCG vaccination [8,29]; secondly, because the presence of NTM in respiratory samples can interfere with the NIH diagnosis of TB as shown in this study.

The distribution of NTM species in this study is similar to what has been reported in South Africa in a collaborative NTM-NET study[30], where *M*. *intracellulare* was the most frequent isolate, followed by *M*. *scrofulaceum* and *gordonae*. Similar to the South African data in the NTM NET study, *M*. *malmoense*, which historically was considered to be restricted to Scandinavia and north-western Europe, was identified in our cohort.

Several challenges are presented when a clinician is managing a suspected case of childhood NTM pulmonary disease. Besides being clinically and radiologically indistinguishable from TB, current guidelines do not provide specific advice for diagnosis of NTM in children. The clinical significance is difficult to establish and often requires longer follow-up and obtainment of several confirmatory samples. In our setting, even if NTM disease was accurately diagnosed in children, treatment is challenging as the availability of rifampicin or ethambutol are limited outside the National TB Program, which only manages tuberculosis cases. Moreover, child friendly formulations are often unavailable and treatment is lengthy and with common side effects[18].

This study had several limitations. Firstly, we do not have a control group to provide data on NTM isolation rates in healthy children nor can we assess the impact of not decontaminating the oral cavity prior to the procedure. Culture negative presumptive TB cases can be a heterogeneous group which can potentially include unidentified culture negative TB. Secondly, only pulmonary samples were obtained and thus, there is no information regarding possible NTM lymphadenitis. Thirdly, as stated before, laboratory contamination, although unlikely, cannot be ruled out completely.

In summary, NTM isolation is frequent in children evaluated for presumed TB in Mozambique and can contribute to overdiagnosing TB. Further studies are needed to understand the epidemiology of NTM in children, including the environmental exposure in other rural Sub-Saharan African settings and the relationship between NTM isolation and BCG efficacy. There is a need for pediatric TB guidelines to discuss the role of NTM isolates and better orient the clinician attending presumptive TB children from high burden TB and low resource countries.
