# Correction: High Rates of Non-Tuberculous Mycobacteria Isolation in Mozambican Children with Presumptive Tuberculosis

**DOI:** 10.1371/journal.pone.0175613

**Published:** 2017-04-06

**Authors:** Elisa López-Varela, Alberto L. García-Basteiro, Orvalho J. Augusto, Oscar Fraile, Helder Bulo, Tasmiya Ira, Kizito Gondo, Jakko van Ingen, Denise Naniche, Jahit Sacarlal, Pedro L. Alonso

The second author’s name appears incorrectly in the citation. The correct citation is: López-Varela E, García-Basteiro AL, Augusto OJ, Fraile O, Bulo H, Ira T, et al. (2017) High Rates of Non-Tuberculous Mycobacteria Isolation in Mozambican Children with Presumptive Tuberculosis. PLoS ONE 12(1): e0169757. doi:10.1371/journal.pone.0169757
